# Some Rare Earth Elements Analysis by Microwave Plasma Torch Coupled with the Linear Ion Trap Mass Spectrometry

**DOI:** 10.1155/2015/156509

**Published:** 2015-09-02

**Authors:** Xiaohong Xiong, Tao Jiang, Wenhao Qi, Jun Zuo, Meiling Yang, Qiang Fei, Saijin Xiao, Aimin Yu, Zhiqiang Zhu, Huanwen Chen

**Affiliations:** ^1^Jiangxi Key Laboratory for Mass Spectrometry and Instrumentation, East China Institute of Technology, Nanchang 330013, China; ^2^College of Chemistry, Jilin University, Changchun 130021, China

## Abstract

A sensitive mass spectrometric analysis method based on the microwave plasma technique is developed for the fast detection of trace rare earth elements (REEs) in aqueous solution. The plasma was produced from a microwave plasma torch (MPT) under atmospheric pressure and was used as ambient ion source of a linear ion trap mass spectrometer (LTQ). Water samples were directly pneumatically nebulized to flow into the plasma through the central tube of MPT. For some REEs, the generated composite ions were detected in both positive and negative ion modes and further characterized in tandem mass spectrometry. Under the optimized conditions, the limit of detection (LOD) was at the level 0.1 ng/mL using MS^2^ procedure in negative mode. A single REE analysis can be completed within 2~3 minutes with the relative standard deviation ranging between 2.4% and 21.2% (six repeated measurements) for the 5 experimental runs. Moreover, the recovery rates of these REEs are between the range of 97.6%–122.1%. Two real samples have also been analyzed, including well and orange juice. These experimental data demonstrated that this method is a useful tool for the field analysis of REEs in water and can be used as an alternative supplement of ICP-MS.

## 1. Introduction

Rare earth elements (REEs), referred to as industrial vitamins, are of particular importance and have extensive applications in various fields, especially in material science and industry, mainly due to their special chemical properties and luminous characters based on their 4f shell electrons [1]. Recently, along with large amounts of exploitation of rare earth resources and the discard of the REEs contained materials, REEs accessed inevitably environment, food chain, and then the human body. Despite this, the positive or negative effect of REEs on the biological body is completely unclear till now. Clearly, a high-sensitive analysis method will promote the researches in this field and give an explicit pattern. Similarly, the potential application fields of REEs are also increasing in current society, and the demand of high-sensitivity analysis methods for REEs is rising gradually. For instance, for biological samples, the sensitivity is general at the level of ng/g. In environmental analysis field, for soil, air, lake water, and so forth, the requirement detection level must at least achieve the magnitude of *μ*g/L, while for sea water and other high technique REEs-containing materials, the according detection level should be the level of ng/L or pg/g [2]. Therefore, developing the sensitive analytical methods for REEs is of vital significance and becomes one of the hot points in analytical science. Because the chemical properties of the 17 REEs are similar, the key point of REEs analysis is the mutual separation of REEs, thus the detection of REEs is usually accompanied by extraction and preseparation [3]. The development of the high-sensitive analytical methods also advances these new separation techniques.

Currently, numerous analytical techniques have been applied in the detection of REEs with the aid of extraction and separation [4], including atomic absorption/fluorescence spectroscopy (AAS/AFS), atomic emission spectroscopy (AES) [5], X-ray fluorescence spectroscopy (XFS) [6], neutron activation analysis (NAA) [7], inductively coupled plasma optical emission spectrometer (ICP-OES) [8], and inductively coupled plasma mass spectrometer (ICP-MS) [9, 10]. Among them, ICP-MS has become one of the most commonly used techniques because of its multiple advantages, including extremely high sensitivity, high analytical speed, broad dynamics range, and synchronous analysis of various elements. The sensitivity of ICP-MS has reached the level of 0.01 ng/g for most REEs and only 10 ng/g for ICP-OES under the same conditions [10]. However, restricted by its large and expensive equipment, the sample injection technique, matrix effect, and the difficulty in the direct analysis of solid sample, ICP-MS is difficult to be applied in the fast field analysis of REEs.

Ambient ionization techniques are a revolution in the mass spectroscopy, which enabled the ionization of samples in their native surrounding with minimum sample pretreatment and brought the breakthrough in the application of MS to high-throughput analysis [11, 12]. There are more than 10 direct ionization techniques based on the atmospheric plasma that have been invented [13], including dielectric barrier discharge ionization (DBDI) [14], desorption atmospheric pressure chemical ionization (DAPCI) [15], desorption corona beam ionization (DCBI) [16], plasma-assisted desorption ionization (PADI) [17], flowing atmospheric pressure afterglow (FAPA) [18, 19], and microplasma discharge ionization (MDI) [20]. Usually, in these methods, the electronic field employed to produce plasma under atmospheric pressure is high discharge voltage (usually several kilovolts) and low frequency (up to 850 Hz) [21]. The temperature of plasma is low (usually several hundred Kelvin in excitation temperature), so that the plasma is weak and even invisible.

The microwave plasma torch (MPT) is a kind of plasma generator at atmospheric pressure by means of the high-frequency (2450 MHz) microwave field, which was developed initially at Jilin University [22], and substantial improvements were made at Indiana University [23]. The MPT can be sustained in a variety of supporting gases, including Ar, He, N_2_, Ne, and air and produce a stable flame-like plasma, similar to ICP. Based on its multiple merits, for instance, small size, low power consumption, low cost, easy operation, and high excitation efficiency, MPT has broad applications in AES portable spectrometer, supercritical fluid chromatography (SFC), and liquid chromatography (LC) as an excitation light source for elemental analysis [24, 25]. Owing to the excellent ionization efficiency, MPT has also been employed as the ion source in mass spectrometry mainly for the elements analysis and the detection of halogenated hydrocarbons separated by a capillary gas chromatography [26]. Recently, Zhang et al. studied the direct desorption/ionization approach of MPT on a linear ion trap (LTQ) mass spectrometer to analyze a series of small organic molecules and showed that MPT is a useful alternative ambient ion source [27]. However, MPT has been rarely used as the detection of metal elements in mass spectrometry. Presumably ICP is dominated in this field. On another aspect, when the analyte is inorganic molecule, the desorption or ionization efficiency is bare due to the ultralow vapor pressure. Moreover, in the usual direct desorption/ionization approach, plasma crossing sideling the sample surface restricts the efficient transformation of the energy from the plasma to the analytes due to the short interaction length. But this obstacle can be overcome by the approach of sample injection via the central tube of MPT. Some previous researches [28] had demonstrated that the MPT-MS is sensitive enough for the demand of field analysis of metal elements in aqueous liquid at the level of 0.02–1 ng/mL.

In this study, an Ar-assisted MPT coupled with the LTQ mass spectrometer was used for the sensitive detection of trace levels of REE ions in water. The nebulized REE aqueous solution, without any pretreatment, flowed into the plasma through the central tube of the MPT to increase the interaction time of the plasma and the samples. For the slat solution of single rare earth elements, including yttrium, lanthanum, cerium, praseodymium, neodymium, samarium, and europium, the generated complex ions in plasma were detected in both positive and negative ion modes and further characterized in collision induced dissociated (CID) experiments. We further examined the semiquantitative capability of this method for the direct detection of REEs in different aqueous liquids.

## 2. Materials and Methods

### 2.1. Materials and Reagents

The 2450 MHz microwave generator (YY1-50 W-2450) and the coaxial line (SFCJ-50-9) were purchased from the Nanjing Electronic Technology Co., Ltd. (Nanjing, China). The MPT was provided by Aimin Yu's group at Jilin University, China. High purity argon (99.999%) was purchased from the Guoteng Special Gas Ltd. (Nanchang, China). LaCl_3_, CeCl_3_, PrCl_3_, EuCl_3_, and NdCl_3_ (analytical grade) were purchased from J&K Chemical Technology Co., Ltd. Yttrium, lanthanum, cerium, praseodymium, neodymium, samarium, and europium standard substance (10^3^ mg/L in 1.0 mol/L HNO_3_) were purchased from Sinopharm Chemical Reagent Co., Ltd. The water used was deionized water provided by the chemistry facilities in the East China Institute of Technology (ECIT). We used pure water and REEs standard substance to prepare a series of solutions with concentrations from 0 ng/mL to 500 ng/mL for the calibration curve measurements. For these REEs, the maximum concentrations for the calibration curve measurements are different based on the practice signal intensities; that is, the linear ranges are discriminating. For instance, for samarium, the maximum concentration is 500 ng/mL, but for yttrium and praseodymium the corresponding values are 100 ng/mL. The practical water sample was gathered in a well in Ganzhou, Jiangxi Province, PR China. Orange juice was gotten by squeezing an orange also produced in Ganzhou. This practical aqueous was directly analyzed with any other pretreatment, except for the necessary dilution with deionized water.

### 2.2. Instrumental Setup

Two mass spectrometers were employed in this study, including a commercial linear ion trap mass spectrometer (Thermo Fisher Scientific, San Jose, CA, USA) and a home-built quadrupole mass filter mass spectrometer. Most of MPT-MS experiments were carried out by the LTQ XL mass spectrometer (see [27]), and the quadrupole mass spectrometer is only used in some assisted experiments. To detect the metal ion in solution, a desolvation unit is combined with the MPT ion source and the LTQ mass spectrometer.

**Figure 1:**
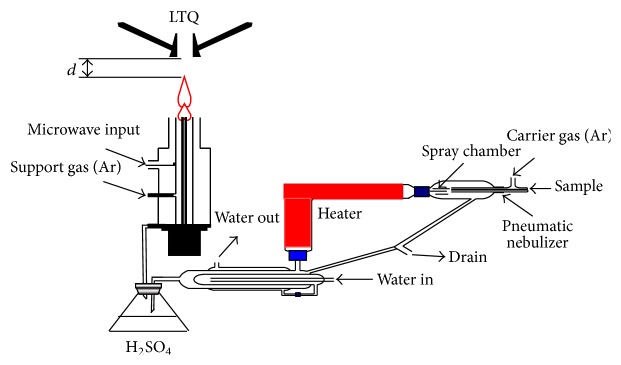
The schematic illustration of the experimental setup including the drying system and MPT.

*The Desolvation Unit.* Water samples, without any pretreatments, were pneumatically nebulized and generate the aerosols. The aerosols flow by carrier gas through a desolvation system to reduce the loss of the microwave power due to the absorption by water. The desolvation system comprised a heated tube and a desiccation chamber. The heated tube is 25 cm long and is wrapped with heating tape which is monitored with a thermocouple and a heater controller (XMT61X, Beijing Huibang Science Technology Co. Ltd., China). The heater controller was set at 140°C ± 5°C. A 500 mL flask was filled with concentrated sulfuric acid (98%) to further remove the H_2_O molecules from the aerosol. At last, the dry aerosols were introduced into the plasma through the central tube of the MPT (see Figure 1). 

*The MPT Ion Source.* The MPT was described previously [29, 30], so only a brief summary of the main features is given here. The MPT consists of three tubes: (1) the outer tube, (2) the intermediate tube, and (3) the central tube. The central tube and the intermediate tube are concentric. The central tube is made of quartz with an outer diameter of 3 mm and inner diameter of 2 mm. The intermediate tube (5.5 mm o.d and 4.5 mm i.d.) and outer tube (26 mm o.d. and 22 mm i.d.) are made of copper. The supporting gas flows in the intermediate tube. And the working gas is divided into two routes, one flows directly in the central tube and the other carries the dried salt in the argon aerosol into the central tube. This structure is called the dual-flow system and is beneficial to optimize the plasma jet shape. Two adjustable rotameters (100–1000 mL/min) are controlled and optimized the two argon flow rates.

A radio-frequency (RF) voltage is applied to the MPT. A 2450-MHz voltage with a maximum power of 100 W is applied to the intermediate tube by the center conductor of a coaxial cable. The shielding ground of the coaxial cable is connected to the outer tube. The RF voltage propagated in the annulus between the intermediate tube and outer tube. The plasma is produced with a spark. A simple tool from a paper clip, a rubber band, and a sheet of A4 paper is employed. The paper clip is bent into a straight line. The paper is wrapped around the paper clip and is secured with the rubber band. About 5 mm of the paper clip extends beyond the clip-paper junction. The remainder of the paper is crumpled to form a handle. The tool was touching the torch between the intermediate tube and the inner tube to create a short circuit between the two tubes, then a cone-shaped plasma jet was generated on the top opening of the MPT. The plasma is stable by adjusting the two gas flow rates. The horizontal distance between the opening end of MPT and the ion inlet of LTQ was about 30 mm.

*The Mass Spectrometer*. The LTQ mass spectrometer was set for the REEs analysis with mainly parameters including the capillary voltage of ±(0–20) V and the tube lens voltage of ±40 V. The temperature of the heated capillary was 150°C. The mass spectra were collected with an average time of 1.5 min. Ions of interest were isolated with a mass-to-charge window width of 0.8 units for collision induced dissociation (CID) experiments by applying a collision energy (CE) of about 30% (arbitrary unit defined by the MS manufacturer). The commercial software Xcalibur is inherently used for LTQ control and data processing.

## 3. Results and Discussion

In general, MPT possesses high excitation efficiency, because the excitation temperature of the plasma is relatively high, about 800–2000 K [31], depending on the microwave power and the working gas. But the ionization degree of MPT is only 0.01%, far less than that of ICP, 0.2% [32]. Thereby some groups with bright characterization can be as reagent ions and make the mass spectra easy assignment. These groups include NO_3_^−^, NH_2_^−^, H_2_O, and OH, since the NO_3_^−^ is electrophilic, while metal ions are general electron donor; the metal element M appears in the MPT mass spectra in the form of M(NO_3_)_*n*_ pulsing several H_2_O molecules or OH groups. Depending on the number *n*, the result group can be cation or anion, so that the REEs can be detected in positive and negative modes. In this study, the characteristic MPT mass spectra of some REEs, including yttrium, lanthanum, cerium, praseodymium, neodymium, samarium, and europium, had been obtained in both positive and negative modes.

### 3.1. MPT Mass Spectra of REEs in Positive Mode

**Figure 2:**
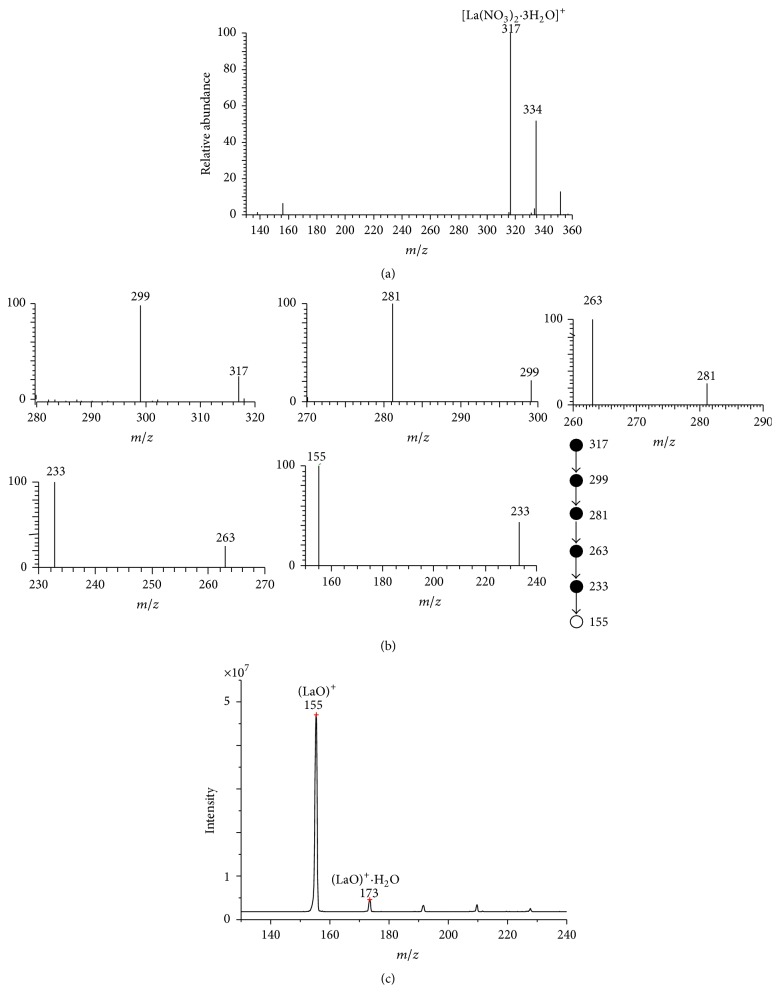
(a) The MPT mass spectrum of lanthanum collected on the MPT-LTQ-MS in positive mode. A 10 mg/L lanthanum standard solution was used. (b) MS^*n*^ mass spectra of the precursor ions *m*/*z* 317, *n* up to 6. The subprecursor ions series are 317 → 299 → 281 → 263 → 233 → 155, corresponding to the dissociation fragment series H_2_O, H_2_O, H_2_O, NO, and NO_4_. (c) The MPT mass spectrum of La collected on a MPT quadrupole mass filer mass spectrometer.

Figure 2(a) showed a typical MPT mass spectrum collected from an experiment using 10 mg/L La standard solution in a full scan positive mode with background subtraction. There are two evidence peaks, located at *m*/*z* 317 and 334. Lanthanum has two natural isotopes: ^139^La with a natural abundance of 99.91% and ^138^La with a natural abundance of 0.89%. The latter is so weak that it is negligible in this MPT spectrum. Combined with the empirical molecular formula mentioned above, the peak at *m*/*z* 317 and 334 can be assigned tentatively to [La(NO_3_)_2_·3H_2_O]^+^ and [La(NO_3_)_2_·3H_2_O·OH]^+^, respectively. To further confirm the assignment of *m*/*z* 317, multistep collision induced dissociation experiments were performed on this peak.  Figure 2(b) showed the MPT MS^*n*^ mass spectra, *n* up to 6, in which the precursor ion *m*/*z* 317 lost successively three H_2_O groups to generate the peak at *m*/*z* 299, 281, and 263 in MS^2^, MS^3^, and MS^4^ spectra, respectively. The subprecursor should contain two NO_3_^−^ groups; in further CID experiments, the main fragment was that of *m*/*z* 233 by the loss of NO in MS^5^ spectrum. The ultimate fragment ion is that of *m*/*z* 155, which was produced from the disaggregation of the ion of *m*/*z* 233 by throwing away a group of NO_4_ and would not be dissociated further in CD experiments. The ion of *m*/*z* 155 may be assigned to (LaO)^+^. Using the LaCl_3_ aqueous solution with the equal concentration, we can get the same results with the final fragment ions of *m*/*z* 155. The results demonstrated again that the relevant anion groups NO_3_^−^ in the MPT mass spectra originated from the background in the plasma but not the sample solution. To further confirm this assignment, we repeated the same experiment on the quadrupole mass filter mass spectrometer with MPT as the ion source; the straightforward ions of *m*/*z* 155 were gained, as shown in Figure 2(c).

It is worthy to pay serious attention to the precursor ion of [La(NO_3_)_2_·3H_2_O]^+^ and [La(NO_3_)_2_·3H_2_O·OH]^+^ in which La element is still kept in its +3 valence state, same as that in its original solution. This is a common character for REEs in MPT-MS, because of the low atomization efficiency in the plasma produced by MPT. Therefore, MPT-MS may gain the information on the original valence states of metallic ions and have a potential as a supplement of ICP-MS. More researches are underway on this aspect.

**Figure 3:**
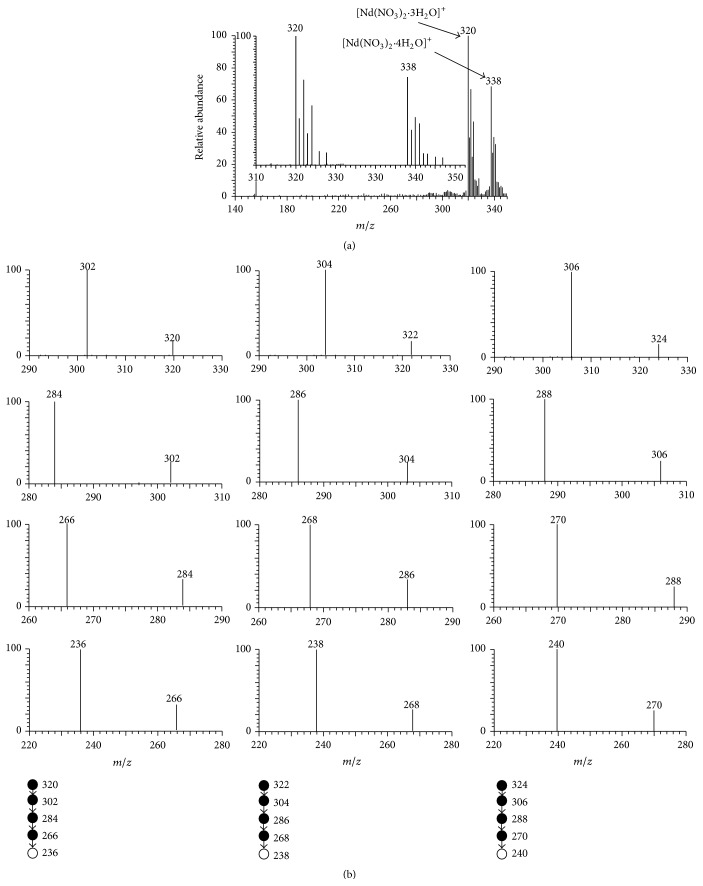
(a) The MPT mass spectrum of neodymium collected on the MPT-LTQ-MS in positive mode. A 10 mg/L neodymium standard solution was used. (b) The MS^2^ to MS^5^ spectra of the ions of *m*/*z* 320, 322, and 324.

Relative to the almost single isotope of lanthanum, neodymium is a representative multiple isotopic element, which has seven major natural isotopes: ^142^Nd with a natural abundance of 27.2%, ^143^Nd with a natural abundance of 12.2%, ^144^Nd with a natural abundance of 23.8%, ^145^Nd with a natural abundance of 8.3%, ^146^Nd with a natural abundance of 17.2%, ^148^Nd with a natural abundance of 5.7%, and ^150^Nd with a natural abundance of 5.6%.  Figure 3 is a typical MPT mass spectrum collected from the experiments of 10 mg/L neodymium standard solution. As shown in Figure 3(a), there were two parallel spectral-shape seven-peak bands with central positions at 320 and 338. Empirically, these two mass spectral bands can be assigned tentatively to [Nd(NO_3_)_2_·3H_2_O]^+^ and [Nd(NO_3_)_2_·4H_2_O]^+^, respectively. Here again, Nd remains in original +3 valence state. The inset in Figure 3 showed the amplified view of the mass spectral region between *m*/*z* 310 and 350. The intensity ratio of major seven peaks at *m*/*z* 320, 321, 322, 323, 324, 326, and 328 was 1 : 0.365 : 0.667 : 0.246 : 0.465 : 0.106 : 0.097, which was integral lower than but yet approximately agreed with the neodymium natural isotopic ratio (1 : 0.448 : 0.875 : 0.305 : 0.632 : 0.209 : 0.206). This approximant contributes to the assignment of [Nd(NO_3_)_2_·3H_2_O]^+^. Although further studies are necessary, one of the possible reasons is the complicated ionization mechanism for metal ions in MPT plasma. In fact, in MPT plasma, the phase transition for metal ions from liquid phase to gas phase may play an important role, as well as the electron collision and redox reactions in plasma. Rigid verification still depended on the tandem mass spectrometry. The MS^*n*^ spectra of these precursor ionic peaks exhibited analogous fragmentation patterns, that is, consecutively losing three H_2_O molecules, NO and NO_4_ to produce (NdO)^+^, analogous to that of La(NO_3_)_2_^+^·3H_2_O. For simplicity, only MS^*n*^ (*n* up to 5) spectra of *m*/*z* 320, 322, and 324 were shown in Figure 3(b), which possesses vivid isotopic characterization. Likewise, similar discussions on the mass spectral band of 338 can give the assignment of [Nd(NO_3_)_2_·4H_2_O]^+^.  Table 1 summarized the dominated peak band in MPT mass spectra of REEs in this study.

### 3.2. MPT Mass Spectra of REEs in Negative Mode

REEs can form the anion in the MPT plasma when the number of the attached NO_3_^−^ exceeds, so that REEs can be detected in negative mode. In fact, the negative mode should be more suitable for the detection of REEs since the negative MPT mass spectra are generally noisier than the positive MPT mass spectra.

**Figure 4:**
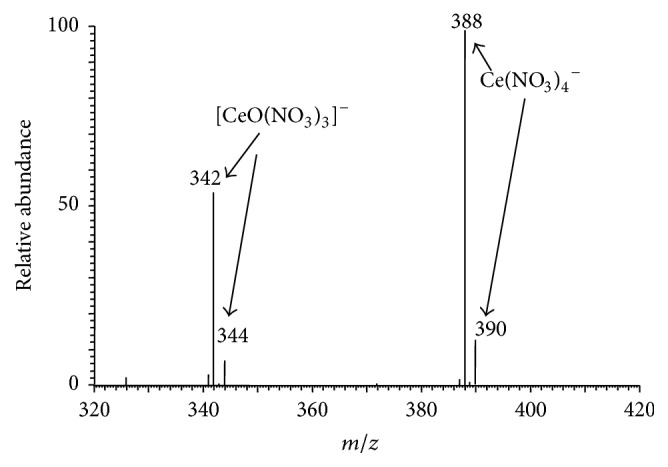
The MPT mass spectrum of cerium collected on the MPT-LTQ-MS in negative mode. A 10 mg/L cerium standard solution was used.

**Figure 5:**
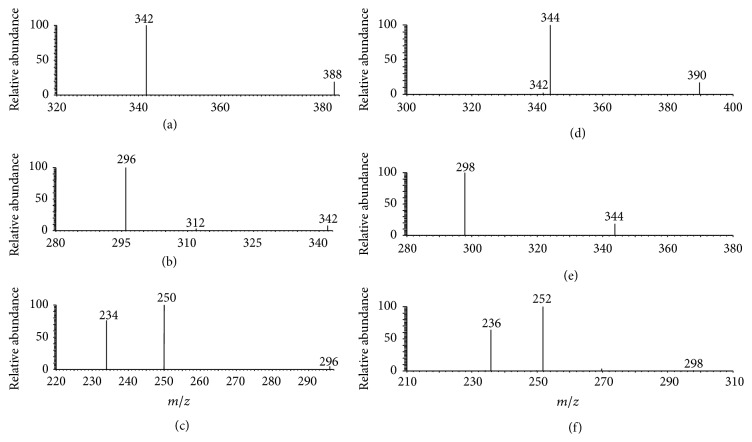
MPT-MS^*n*^ mass spectra of precursor anions of *m*/*z* 388 and 390 (*n* up to 4).

Figure 4 is the typical MPT mass spectrum of cerium in negative mode collected in the range of *m*/*z* 320–420, where a 10 mg/L cerium standard solution was used. Clearly, there are two pairs of peak, *m*/*z* 342 and 344 as well as *m*/*z* 388 and 390, with intensity ratio of 7.65 and 8. Cerium has four natural isotopes, ^136^Ce(0.19%), ^138^Ce(0.25%), ^140^Ce(88.48%), and ^142^Ce(11.07%). Ignoring the former two isotopes since their abundance is too small, the abundance ratio of the latter two isotopes is 7.99, which nearly equals the intensity ratio of the two peaks in latter pair. Based on the fine matching, combined with the empirical molecular formula, the ion of *m*/*z* 342 can be assigned to [^140^CeO(NO_3_)_3_]^−^ and the ion of *m*/*z* 388 is [^140^Ce(NO_3_)_4_]^−^. The companions are the corresponding isotopic ions. It is easy to see that the structure of [Ce(NO_3_)_4_]^−^ is more symmetric so that the signals are more intense and the intensity ratio is much closer to the theoretical values. Analogously, the MPT tandem mass spectrometry in negative mode also provides partial evidences about the structure of the REEs anions. Figures 5(a)–5(c) are the MS^*n*^ mass spectra of the precursor ions at *m*/*z* 388, *n* = 2, 3, 4, respectively. As shown, the precursor ions of *m*/*z* 388 produce first the fragment of *m*/*z* 342 by losing a NO_2_ group. In MS^3^, subprecursor of *m*/*z* 342 can produce the minor fragment of *m*/*z* 312 by the loss of NO group and the major fragment of *m*/*z* 296 by throwing away a second NO_2_ group, and the fragment of *m*/*z* 296 yields the peak at *m*/*z* 250 by the loss of a third NO_2_ group and yields the peak at *m*/*z* 234 by losing a NO_3_ group. The ions of *m*/*z* 234 would dissociate further under current experimental conditions possibly because the signal intensity in MS^4^ is low. However, sequential loss of 46-D group (NO_2_) hints that the precursor ion of *m*/*z* 388 is polynitrogen complex. Figures 5(d)–5(f) depict the tandem mass spectra of the ions of *m*/*z* 390 showing parallel isotopic pattern. Thus combined with the abundant ration, the fact that the ion of *m*/*z* 388 was assigned to [Ce(NO_3_)_4_]^−^ is reasonable. The characteristic MPT anionic peaks of seven REEs in this study were also summarized in Table 1.

### 3.3. Direct and Semiquantitative Measurement Using MPT-MS

MPT-LTQ MS in negative running mode is less noisier and more efficient than that in positive mode, and the quantitative performance of MPT-LTQ negative MS is better than that of MPT-LTQ positive MS in this study. Therefore, we adopted the negative mode to measure semiquantitatively the concentration of single REE in water to verify the capacity of MPT-MS in the detection of REEs. In addition, to reduce the possibility of a false signal originated from the isotopes of adjacent REES, the major fragments in the MPT-MS^2^ mass spectra were proposed as the signal for the qualification of REEs in water samples. The linear relationships between the measured fragment signal intensities and the concentrations of each spiked REE in water were measured with the concentration range of 0 to several hundred ppb (ng/mL), at least two orders of magnitude, with the determination coefficient *R* ^2^ larger than 0.99. To be noticed, for each data point, six measurements were repeated with the relative standard deviation (RSD) almost less than 15%. Only some data of samarium are a little beyond 20%. The LOD of this method were estimated at the level of 0.1 ng/mL according to triple noise level at the lowest concentration. The LOD for praseodymium is even as low as 0.04 ng/mL, almost close to the level of ICP-MS [2, 10]. The largest values of LOD appear in yttrium, the only nonlanthanide REEs in this study, arriving at 0.5 ng/mL. In addition, adding recovery experiments (for almost all REEs in this study, 5 ng/mL solutions were used, but for yttrium 7.5 ng/mL solution was used) also give the recovery rates of these seven REEs in a reasonable interval of 97.6%–122.1%. The detailed data were all summarized in Table 2. Totally, although these LOD values are higher about one magnitude than those obtained in ICP-MS, this MPT-LTQ-MS is still valuable in particular applications as an alternative supplement of ICP-MS for the field analysis of REEs in water. As a primary application, according to these calibration curves, the two practical aqueous samples were directly analyzed to obtain the content of these seven REEs. The analysis results were also given in Table 2.

## 4. Conclusions

Conclusively, a sensitive method based on MPT-LTQ MS has been developed to determine individual content of some REEs in water at trace levels, including yttrium, lanthanum, cerium, praseodymium, neodymium, samarium, and europium. By means of tandem mass spectrometry and isotopic abundant ratio, characteristic MPT mass spectra of these seven REEs were identified both in positive and negative modes; meanwhile the main fragment signals in MS^2^ mass spectra were also quantitative measurement. The results showed that this MPT-MS^2^ method has enough sensitivity to determine trace levels of these seven REEs in water with the LOD values at ~0.1 ng/mL level with reasonable semiquantitative performance. Therefore, this method has potential applications in quality monitoring of single REE in water. Nevertheless, in real applications, this MPT-MS ought to combine with some extraction and separation methods to isolate single REE for accurate analysis. However, as a low-cost and sensitive method, it is still promising and can be used as a supplement of ICP applied in field analysis of single REE in water.

## Figures and Tables

**Table 1:** Characteristic peaks of REEs in MPT-MS.

REEs	Natural isotopes (%)	Dominant peak band in MPT-MS (*m*/*z*)		Normalized intensity of corresponding peaks		Assignment	
		+mode^a^	−mode^b^	+mode	−mode	+mode	−mode
Y	^89^Y (100)	267	337	1	1	[Y(NO_3_)_2_·3H_2_O]^+^	Y(NO_3_)_4_^−^

La	^138^La (0.09)	—	—	—	—	[La(NO_3_)_2_·3H_2_O]^+^	La(NO_3_)_4_^−^
	^139^La (99.91)	317	387	1	1		

Ce	^136^Ce (0.19)	—	—	—	—	[Ce(NO_3_)_2_·3H_2_O]^+^	Ce(NO_3_)_4_^−^
	^138^Ce (0.25)	—	—	—	—		
	^140^Ce (88.48)	318	388	1	1		
	^142^Ce (11.08)	320	390	0.112	0.1205		

Pr	^141^Pr (100)	337	389	1	1	[Pr(NO_3_)_2_·4H_2_O]^+^	Pr(NO_3_)_4_^−^

Nd	^142^Nd (27.13)	320	390	1	1	[Nd(NO_3_)_2_·3H_2_O]^+^	Nd(NO_3_)_4_^−^
	^143^Nd (12.18)	321	391	0.419	0.458		
	^144^Nd (23.80)	322	392	0.8401	0.8714		
	^145^Nd (8.30)	323	393	0.3002	0.325		
	^146^Nd (17.19)	324	394	0.624	0.638		
	^148^Nd (5.76)	326	396	0.181	0.2247		
	^150^Nd (5.64)	328	398	0.1712	0.217		

Sm	^144^Sm (3.1)	322	392	0.138	0.1253	[Sm(NO_3_)_2_·3H_2_O]^+^	Sm(NO_3_)_4_^−^
	^147^Sm (15.0)	325	395	0.5887	0.5767		
	^148^Sm (11.3)	326	396	0.4112	0.4368		
	^149^Sm (13.8)	327	397	0.501	0.5395		
	^150^Sm (7.4)	328	398	0.2562	0.2911		
	^152^Sm (26.7)	330	400	1	1		
	^154^Sm (22.7)	332	402	0.8472	0.8318		

Eu	^151^Eu (47.8)	329	399	0.916	0.9148	[Eu(NO_3_)_2_·3H_2_O]^ +^	Eu(NO_3_)_4_^−^
	^153^Eu (52.2)	331	401	1	1		

^a^10 mg/L standard solutions were used.

^b^0.5 mg/L standard solutions were used.

**Table 2:** Quantitative indexes summarized of MPT-MS^2^ method for REEs in pure water and analysis results for some real samples.

REEs	Linear equation	*R* ^2^	Linear range (ng/mL)	LOD (ng/mL)	RSD (*n* = 6, %)	Recovery rate	Well water (ng/mL)	Orange juice (ng/mL)
Y	*y* = 29.105 × *c* − 48.27	0.999	1–100	0.574	3–11	103.4^a^	31.41	135.45
La	*y* = 3.158 × *c* − 11.205	0.998	1–250	0.2	7.8–14.1	117.3^b^	0.759	20.33
Ce	*y* = 9.073 × *c* − 24.562	0.998	1–250	0.29	6.3–9.4	122.1^b^	1.365	3
Pr	*y* = 24.890 × *c* − 10.879	0.999	1–100	0.04	2.4–10.1	100.5^b^	0.47	0.77
Nd	*y* = 7.731 × *c* + 4.463	0.998	1–250	0.114	3.6–14.7	113.7^b^	6.38	1.94
Sm	*y* = 0.929 × *c* − 7.541	0.998	1–500	0.388	9.4–21.2	109.7^b^	0.98	396.27
Eu	*y* = 2.039 × *c* − 5.694	0.999	1–250	0.369	3–8.1	97.6^b^	—	0.725

^a^7.5 ng/mL.

^b^5 ng/mL.

## References

[1] Xu G. *Rare Earths, a Three-Volume Comprehensive Monograph of the Science and Technology of Rare Earths*.

[2] Hu B., Yin J. (2006). Progress of separation and determination methods for rare earth elements. *Journal of the Chinese Rare Earth Society*.

[3] Rao T. P., Kala R. (2004). On-line and off-line preconcentration of trace and ultratrace amounts of lanthanides. *Talanta*.

[4] He M., Hu B., Jiang Z. Y. (2005). Progress of the analytical method for rare earth elements determination. *Journal of Analytical Science*.

[5] Jiang W., Ma Y. D., Zhao W. Y., Feng Y., Wang N. X., Si Z. K. (2003). Determination of trace europium by use of the new fluorescence system europium-sparfloxacin-1,10-phenanthroline-sodium dodecyl sulfate. *Analytical and Bioanalytical Chemistry*.

[6] Fernández-Ruiz R., Capmany J. (2001). Determination of the rare-earth: Nb mass ratio in doped LiNbO_3_ by the TXRF technique. *Journal of Analytical Atomic Spectrometry*.

[7] Ohde S. (2003). Determination of rare earth elements in carbonatites from the Kangankunde Mine, Malawi by instrumental neutron activation analysis. *Journal of Radioanalytical and Nuclear Chemistry*.

[8] Yenisoy-Karakaş S., Gaga E. O., Doğangün A., Tuncel S. G. (2004). Determination of major and rare earth elements in bastnasite ores by ICP-AES. *Analytical Letters*.

[9] Moor C., Devos W., Guecheva M., Kobler J. (2000). Inductively coupled plasma mass spectrometry: a versatile tool for a variety of different tasks. *Fresenius' Journal of Analytical Chemistry*.

[10] Balaram V. (1996). Recent trends in the instrumental analysis of rare earth elements in geological and industrial materials. *TrAC—Trends in Analytical Chemistry*.

[11] Cooks R. G., Ouyang Z., Takats Z., Wiseman J. M. (2006). Ambient mass spectrometry. *Science*.

[12] Huang M.-Z., Cheng S.-C., Cho Y.-T., Shiea J. (2011). Ambient ionization mass spectrometry: a tutorial. *Analytica Chimica Acta*.

[13] Harris G. A., Galhena A. S., Fernández F. M. (2011). Ambient sampling/ionization mass spectrometry: applications and current trends. *Analytical Chemistry*.

[14] Na N., Zhao M., Zhang S., Yang C., Zhang X. R. (2007). Development of a dielectric barrier discharge ion source for ambient mass spectrometry. *Journal of the American Society for Mass Spectrometry*.

[15] Yang S. P., Ding J. H., Zheng J. Q. (2009). Detection of melamine in milk products by surface desorption atmospheric pressure chemical ionization mass spectrometry. *Analytical Chemistry*.

[16] Li X., Wang H., Sun W., Ding L. (2010). Desorption corona beam ionization coupled with a poly(dimethylsiloxane) substrate: broadening the application of ambient ionization for water samples. *Analytical Chemistry*.

[17] Ratcliffe L. V., Rutten F. J. M., Barrett D. A. (2007). Surface analysis under ambient conditions using plasma-assisted desorption/ionization mass spectrometry. *Analytical Chemistry*.

[18] Andrade F. J., Shelley J. T., Wetzel W. C. (2008). Atmospheric pressure chemical ionization source. 2. Desorption-ionization for the direct analysis of solid compounds. *Analytical Chemistry*.

[19] Andrade F. J., Shelley J. T., Wetze W. C. (2008). Atmospheric pressure chemical ionization source. 1. Ionization of compounds in the gas phase. *Analytical Chemistry*.

[20] Symond J. M., Galhena A. S., Fernández F. M., Orlando T. M. (2010). Microplasma discharge ionization source for ambient mass spectrometry. *Analytical Chemistry*.

[21] Taghioskoui M., Zaghloul M., Montaser A. An atmospheric pressure ultrahigh frequency plasma jet for ambient mass spectrometry.

[22] Jin Q., Yang G., Yu A., Liu J., Zhang H., Ben Y. (1985). A novel plasma emission source. *Natural Science Journal of Jilin University*.

[23] Jin Q., Chu Z., Borer M. W., Hieftje G. M. (1991). Microwave plasma torch assembly for atomic emission spectrometry. *Spectrochimica Acta Part B: Atomic Spectroscopy*.

[24] Feng G., Huan Y., Cao Y. (2004). Development of a miniature simultaneous MPT spectrometer. *Microchemical Journal*.

[25] Duan Y., Su Y., Jin Z., Abeln S. P. (2000). Design and development of a highly sensitive, field portable plasma source instrument for on-line liquid stream monitoring and real-time sample analysis. *Review of Scientific Instruments*.

[26] Pack B. W., Broekaert J. A. C., Guzowski J. P., Poehlman J., Hieftje G. M. (1998). Determination of halogenated hydrocarbons by helium microwave plasma torch time-of-flight mass spectrometry coupled to gas chromatography. *Analytical Chemistry*.

[27] Zhang T. Q., Zhou W., Jin W. (2013). Direct desorption/ionization of analytes by microwave plasma torch for ambient mass spectrometric analysis. *Journal of Mass Spectrometry*.

[28] Xiong X. H., Luo Y. L., Jiang T. (2015). Studies of microwave plasma torch mass spectra of manganese, iron, cobalt, nickel, copper, and zinc in negative mode. *Journal of Instrumental Analysis*.

[29] Yang W., Zhang H., Yu A., Jin Q. (2000). Microwave plasma torch analytical atomic spectrometry. *Microchemical Journal*.

[30] Barnes J. H., Grøn O. A., Hieftje G. M. (2002). Characterization of an argon microwave plasma torch coupled to a Mattauch-Herzog geometry mass spectrometer. *Journal of Analytical Atomic Spectrometry*.

[31] Wang S. H. (2006). *Studies on the diagnoses and application of oxygen-shielded argon microwave plasma torch (OS-ArMPT) [Ph.D. thesis]*.

[32] Timmermans E. (1999). *Atomic and molecular excitation processes in microwave induced plasmas, a spectroscopic study [M.S. thesis]*.

